# Di-μ-iodido-bis­(iodido­{methyl 4-[(pyridin-2-yl­methyl­idene)amino]­benzoate-κ^2^ *N*,*N*′}cadmium)

**DOI:** 10.1107/S160053681302905X

**Published:** 2013-10-31

**Authors:** Tushar S. Basu Baul, Sajal Kundu, Seik Weng Ng, Edward R. T. Tiekink

**Affiliations:** a Department of Chemistry, North-Eastern Hill University, NEHU Permanent Campus, Umshing, Shillong 793 022, India; b Department of Chemistry, University of Malaya, 50603 Kuala Lumpur, Malaysia; c Chemistry Department, Faculty of Science, King Abdulaziz University, PO Box 80203 Jeddah, Saudi Arabia

## Abstract

The complete binuclear molecule of the title compound, [Cd_2_I_4_(C_14_H_12_N_2_O_2_)_2_], is generated by the application of a centre of inversion. The Cd—I bond lengths of the central core are close and uniformly longer than the exocyclic Cd—I bond. The coordination sphere of the Cd^II^ atom is completed by two N atoms of a chelating methyl 4-[(pyridin-2-yl­methyl­idene)amino]­benzoate ligand, and is based on a square pyramid with the terminal I atom in the apical position. The three-dimensional crystal packing is stabilized by C—H⋯O and C—H⋯π inter­actions, each involving the pyridine ring.

## Related literature

For spectroscopic, biological and structural studies of zinc triad elements with (*E*)-*N*-(pyridin-2-yl­methyl­idene)aryl­amine ligands, see: Basu Baul, Kundu, Höpfl *et al.* (2013 ▶); Basu Baul, Kundu, Linden *et al.* (2013 ▶); Basu Baul, Kundu, Mitra *et al.* (2013 ▶). For additional structural analysis, see: Addison *et al.* (1984 ▶).
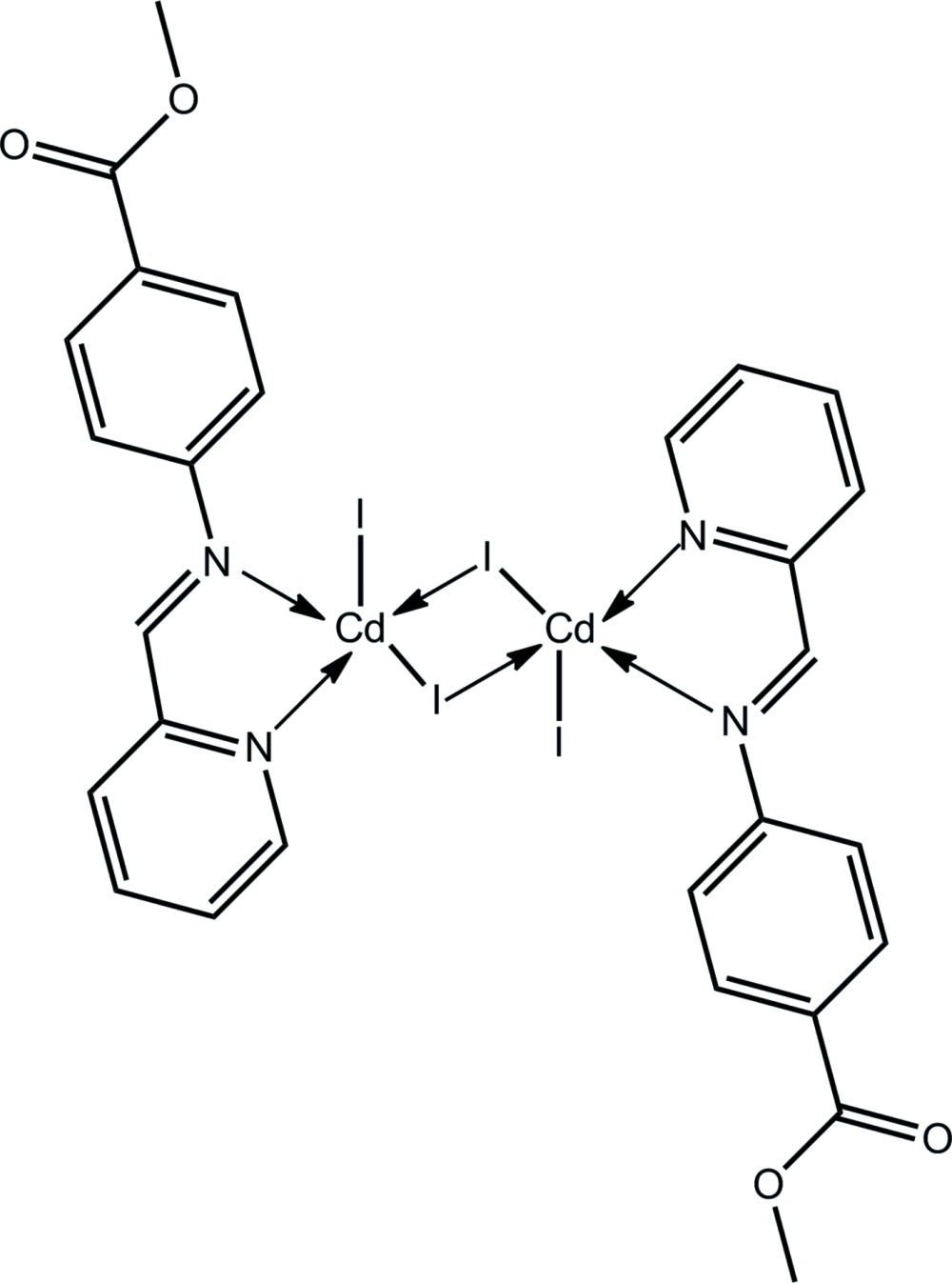


## Experimental

### 

#### Crystal data

[Cd_2_I_4_(C_14_H_12_N_2_O_2_)_2_]
*M* *_r_* = 1212.91
Triclinic, 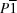

*a* = 8.4883 (3) Å
*b* = 9.3677 (5) Å
*c* = 10.9029 (5) Å
α = 109.516 (5)°
β = 95.868 (3)°
γ = 90.242 (4)°
*V* = 812.18 (6) Å^3^
*Z* = 1
Mo *K*α radiation
μ = 5.15 mm^−1^
*T* = 100 K
0.20 × 0.15 × 0.10 mm

#### Data collection

Agilent SuperNova Dual diffractometer with an Atlas detector
Absorption correction: multi-scan (*CrysAlis PRO*; Agilent, 2013 ▶) *T*_min_ = 0.698, *T*_max_ = 1.000
11886 measured reflections
3738 independent reflections
3374 reflections with *I* > 2σ(*I*)
*R*_int_ = 0.031

#### Refinement

*R*[*F* ^2^ > 2σ(*F* ^2^)] = 0.022
*wR*(*F* ^2^) = 0.050
*S* = 1.00
3738 reflections
191 parameters
H-atom parameters constrained
Δρ_max_ = 0.58 e Å^−3^
Δρ_min_ = −1.04 e Å^−3^

### 

Data collection: *CrysAlis PRO* (Agilent, 2013 ▶); cell refinement: *CrysAlis PRO*; data reduction: *CrysAlis PRO*; program(s) used to solve structure: *SHELXS97* (Sheldrick, 2008 ▶); program(s) used to refine structure: *SHELXL97* (Sheldrick, 2008 ▶); molecular graphics: *ORTEP-3 for Windows* (Farrugia, 2012 ▶) and *DIAMOND* (Brandenburg, 2006 ▶); software used to prepare material for publication: *publCIF* (Westrip, 2010 ▶).

## Supplementary Material

Crystal structure: contains datablock(s) general, I. DOI: 10.1107/S160053681302905X/hg5357sup1.cif

Structure factors: contains datablock(s) I. DOI: 10.1107/S160053681302905X/hg5357Isup2.hkl

Additional supplementary materials:  crystallographic information; 3D view; checkCIF report

## Figures and Tables

**Table 1:** Selected bond lengths (Å)

I1—Cd	2.8765 (4)
I1—Cd^i^	2.9813 (3)
I2—Cd	2.7023 (4)
Cd—N1	2.333 (2)
Cd—N2	2.402 (2)

Symmetry code: (i) 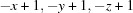.

**Table 2:** Hydrogen-bond geometry (Å, °) *Cg*1 is the centroid of the N1,C1–C5 ring.

*D*—H⋯*A*	*D*—H	H⋯*A*	*D*⋯*A*	*D*—H⋯*A*
C1—H1⋯O2^ii^	0.95	2.34	3.066 (4)	133
C14—H14*B*⋯*Cg*1^iii^	0.98	2.79	3.416 (4)	123

Symmetry codes: (ii) 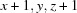; (iii) 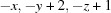.

**Fig. 1.**
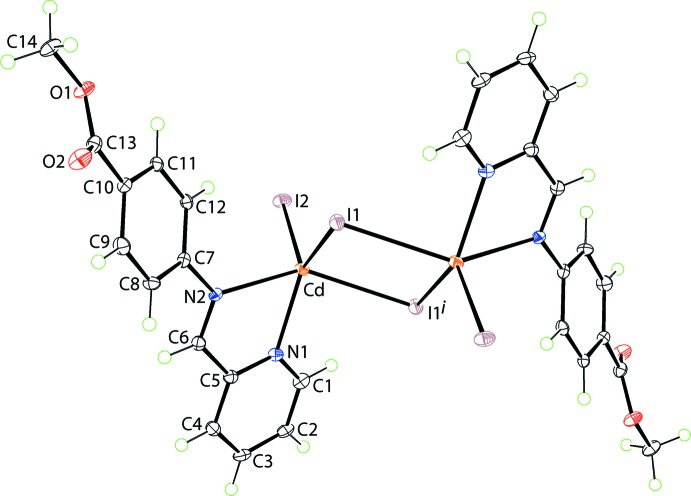
Molecular structure of the centrosymmetric binuclear molecule of (I) showing atom-labelling scheme and displacement ellipsoids at the 50% probability level. The primed atom is related by the symmetry operation 1 - x, 1 - y, 1 - z.

**Fig. 2.**
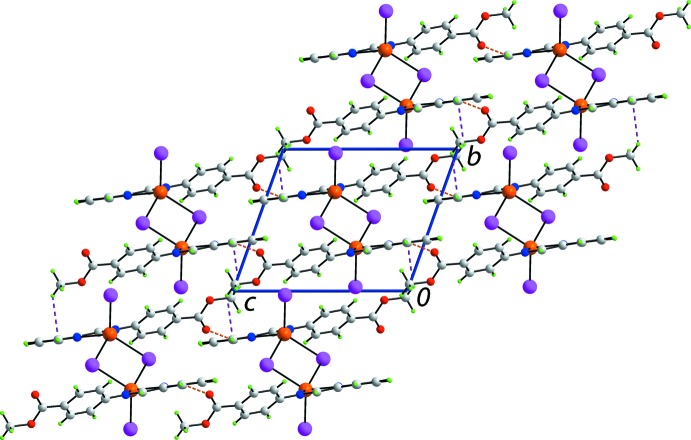
A view of the unit-cell contents in projection down the a axis in (I). The C—H···O and C—H···π interactions are shown as orange and purple dashed lines, respectively.

## supplementary crystallographic information

### 1. Comment

The title compound, (I), was investigated during the course of studies into the coordination chemistry of divalent zinc triad elements with (*E*)-*N*-(pyridin-2-ylmethylidene)arylamine ligands. These complexes were investigated primarily by X-ray crystallography and proton NMR but, also included some biological studies (Basu Baul, Kundu, Höpfl *et al.*, 2013; Basu Baul, Kundu, Linden *et al.*, 2013; Basu Baul, Kundu, Mitra *et al.* 2013).

The centrosymmetric binuclear compound, Fig. 1, features a central Cd_2_I_2_ core that approximates a square as the µ_2_-I atoms form almost equivalent Cd—I bond lengths, each of which is longer than the terminal Cd—I bond, Table 1. The five-coordinate environment is completed by the chelating ligand which exhibits a twist as seen in the dihedral angle between the two rings of 30.78 (17)°. The coordination geometry approximates a square pyramid as judged by the value of τ = 0.13, compared with 0.0 and 1.0 for ideal square pyramidal and trigonal bipyramidal geometries, respectfully (Addison *et al.*, 1984). In this description, the Cd atom lies 0.9208 (1) Å above the plane defined by the two µ_2_-I and chelating N atoms (r.m.s. deviation = 0.0690 Å) in the direction of the terminal I atom. As observed in related systems, the Cd—(pyridyl) bond length is shorter than the Cd—*N*(imino) bond. The ester group is twisted out of the plane of the benzene ring to which it is connected as seen in the value of the C9—C10—C13—O1 torsion angle of 161.5 (3)°.

The crystal packing is dominated by interactions involving the pyridyl residue, Table 2. Thus, pyridyl-*C*—*H*···*O*(carbonyl) and methyl-H···π(pyridyl) interaction stabilize the three-dimensional architecture, Fig. 2.

The binuclear structure reported herein contrasts the mononuclear structures found for the zinc (Basu Baul, Kundu, Linden *et al.*, 2013) and mercury (Basu Baul, Kundu, Mitra *et al.*, 2013) analogues.

### 2. Experimental

To a solution of pyridine-2-carboxaldehyde (0.10 g, 0.93 mmol) in ethanol (3 ml) was added a solution of methyl *p*-aminobenzoate (0.15 g, 0.99 mmol) in ethanol (2 ml). The mixture was stirred at ambient temperature for 30 min. To this reaction mixture, a solution of CdI_2_ (0.36 g, 0.98 mmol) in methanol (20 ml) was added drop-wise under stirring conditions which resulted in the immediate formation of a yellow precipitate. The stirring was continued for 3 h and then the mixture was filtered. The residue was washed with methanol (3 *x* 5 ml) and dried *in vacuo*. The dried solid was dissolved by boiling in acetonitrile (40 ml) and filtered while hot. The filtrate, upon cooling to r.t., afforded yellow crystalline material. Yield 43%. *M*. pt:. 511–513 K. Analysis, calculated for C_14_H_12_CdI_2_N_2_O_2_: C, 27.71, H, 1.99, N, 4.62%; Found: C, 27.82; H, 2.05; N, 4.67%. Λ_m_(CH_3_CN): 7 Ω^-1^cm^2^mol^-1^. IR (cm^-1^):1717 ν*_asym_*(OCO), 1591 ν*_asym_*(C(H)═N). ^1^H-NMR (DMSO-d_6_; refer to Fig. 1 for atom numbering): 9.14 [d, J = 4.5 Hz, 1H, H-1], 8.73 [s, 1H, H-6], 8.16 [t, br, 1H, H-4], 8.03 [m, 3H, H-3,9,11], 7.80 [t, br, 1H, H-2], 7.57 [d, J = 8.0 Hz, 2H, H-8,12], 3.91 [s, 3H, H-14] p.p.m.. Crystals of compound suitable for X-ray crystal-structure determination were obtained from acetonitrile/chloroform by slow evaporation of the solvent at room temperature.

### 3. Refinement

Carbon-bound H-atoms were placed in calculated positions [C—H 0.95 to 0.98 Å, *U*_iso_(H) 1.2 to 1.5*U*_eq_(C)] and were included in the refinement in the riding model approximation. Two reflections, *i.e*. (1 0 0) and (-5 0 2), were omitted from the final refinement owing to poor agreement. The maximum and minimum residual electron density peaks of 0.58 and 1.04 e Å^-3^, respectively, were located 0.69 Å and 0.49 Å from the I2 and I1 atoms, respectively.

### Crystal data

**Table:**

[Cd_2_I_4_(C_14_H_12_N_2_O_2_)_2_]	*Z* = 1
*M**_r_* = 1212.91	*F*(000) = 560
Triclinic, *P*1	*D*_x_ = 2.480 Mg m^−^^3^
Hall symbol: -P 1	Mo *K*α radiation, λ = 0.71073 Å
*a* = 8.4883 (3) Å	Cell parameters from 5933 reflections
*b* = 9.3677 (5) Å	θ = 2.3–27.5°
*c* = 10.9029 (5) Å	µ = 5.15 mm^−^^1^
α = 109.516 (5)°	*T* = 100 K
β = 95.868 (3)°	Prism, yellow
γ = 90.242 (4)°	0.20 × 0.15 × 0.10 mm
*V* = 812.18 (6) Å^3^	

### Data collection

**Table:**

Agilent SuperNova Dual diffractometer with an Atlas detector	3738 independent reflections
Radiation source: SuperNova (Mo) X-ray Source	3374 reflections with *I* > 2σ(*I*)
Mirror monochromator	*R*_int_ = 0.031
Detector resolution: 10.4041 pixels mm^-1^	θ_max_ = 27.6°, θ_min_ = 2.3°
ω scan	*h* = −10→11
Absorption correction: multi-scan (*CrysAlis PRO*; Agilent, 2013)	*k* = −12→12
*T*_min_ = 0.698, *T*_max_ = 1.000	*l* = −14→14
11886 measured reflections	

### Refinement

**Table:**

Refinement on *F*^2^	Primary atom site location: structure-invariant direct methods
Least-squares matrix: full	Secondary atom site location: difference Fourier map
*R*[*F*^2^ > 2σ(*F*^2^)] = 0.022	Hydrogen site location: inferred from neighbouring sites
*wR*(*F*^2^) = 0.050	H-atom parameters constrained
*S* = 1.00	*w* = 1/[σ^2^(*F*_o_^2^) + (0.021*P*)^2^ + 0.5485*P*] where *P* = (*F*_o_^2^ + 2*F*_c_^2^)/3
3738 reflections	(Δ/σ)_max_ = 0.001
191 parameters	Δρ_max_ = 0.58 e Å^−^^3^
0 restraints	Δρ_min_ = −1.04 e Å^−^^3^

### Special details

**Table:**

Geometry. All e.s.d.'s (except the e.s.d. in the dihedral angle between two l.s. planes) are estimated using the full covariance matrix. The cell e.s.d.'s are taken into account individually in the estimation of e.s.d.'s in distances, angles and torsion angles; correlations between e.s.d.'s in cell parameters are only used when they are defined by crystal symmetry. An approximate (isotropic) treatment of cell e.s.d.'s is used for estimating e.s.d.'s involving l.s. planes.
Refinement. Refinement of *F*^2^ against ALL reflections. The weighted *R*-factor *wR* and goodness of fit *S* are based on *F*^2^, conventional *R*-factors *R* are based on *F*, with *F* set to zero for negative *F*^2^. The threshold expression of *F*^2^ > σ(*F*^2^) is used only for calculating *R*-factors(gt) *etc*. and is not relevant to the choice of reflections for refinement. *R*-factors based on *F*^2^ are statistically about twice as large as those based on *F*, and *R*- factors based on ALL data will be even larger.

### Fractional atomic coordinates and isotropic or equivalent isotropic displacement parameters (Å^2^)

**Table:**

	*x*	*y*	*z*	*U*_iso_*/*U*_eq_	
I1	0.36407 (2)	0.52109 (2)	0.344025 (19)	0.01447 (6)	
I2	0.54922 (2)	0.97175 (3)	0.69338 (2)	0.01683 (6)	
Cd	0.39702 (2)	0.69721 (3)	0.61836 (2)	0.01149 (6)	
N1	0.2891 (3)	0.6752 (3)	0.8000 (2)	0.0118 (5)	
N2	0.1161 (3)	0.7293 (3)	0.5962 (2)	0.0110 (5)	
O1	−0.1491 (3)	0.9351 (3)	0.1141 (2)	0.0162 (5)	
O2	−0.3079 (3)	0.7290 (3)	0.0766 (2)	0.0206 (5)	
C1	0.3737 (4)	0.6588 (4)	0.9039 (3)	0.0153 (7)	
H1	0.4858	0.6579	0.9063	0.018*	
C2	0.3043 (4)	0.6428 (4)	1.0096 (3)	0.0150 (7)	
H2	0.3682	0.6340	1.0833	0.018*	
C3	0.1418 (4)	0.6401 (4)	1.0051 (3)	0.0157 (7)	
H3	0.0915	0.6254	1.0741	0.019*	
C4	0.0520 (4)	0.6593 (4)	0.8977 (3)	0.0139 (7)	
H4	−0.0604	0.6582	0.8924	0.017*	
C5	0.1304 (4)	0.6800 (4)	0.7980 (3)	0.0123 (6)	
C6	0.0422 (4)	0.7113 (4)	0.6870 (3)	0.0119 (6)	
H6	−0.0697	0.7181	0.6826	0.014*	
C7	0.0294 (3)	0.7576 (4)	0.4877 (3)	0.0107 (6)	
C8	−0.1235 (4)	0.6954 (4)	0.4383 (3)	0.0130 (6)	
H8	−0.1757	0.6374	0.4800	0.016*	
C9	−0.1987 (4)	0.7191 (4)	0.3279 (3)	0.0153 (7)	
H9	−0.3021	0.6761	0.2933	0.018*	
C10	−0.1227 (3)	0.8059 (4)	0.2677 (3)	0.0103 (6)	
C11	0.0284 (3)	0.8706 (4)	0.3191 (3)	0.0115 (6)	
H11	0.0785	0.9326	0.2798	0.014*	
C12	0.1058 (3)	0.8445 (4)	0.4279 (3)	0.0114 (6)	
H12	0.2101	0.8857	0.4613	0.014*	
C13	−0.2045 (4)	0.8179 (4)	0.1441 (3)	0.0135 (7)	
C14	−0.2324 (4)	0.9537 (4)	−0.0024 (3)	0.0175 (7)	
H14A	−0.2363	0.8575	−0.0752	0.026*	
H14B	−0.1767	1.0318	−0.0248	0.026*	
H14C	−0.3406	0.9843	0.0144	0.026*	

### Atomic displacement parameters (Å^2^)

**Table:**

	*U*^11^	*U*^22^	*U*^33^	*U*^12^	*U*^13^	*U*^23^
I1	0.01401 (11)	0.01747 (12)	0.01205 (11)	0.00245 (8)	0.00116 (8)	0.00518 (9)
I2	0.01733 (11)	0.01644 (12)	0.01742 (11)	−0.00593 (9)	−0.00164 (8)	0.00774 (9)
Cd	0.01011 (11)	0.01394 (13)	0.01083 (12)	−0.00150 (9)	0.00068 (9)	0.00488 (10)
N1	0.0126 (13)	0.0116 (14)	0.0107 (13)	−0.0020 (11)	0.0000 (10)	0.0034 (11)
N2	0.0121 (12)	0.0113 (14)	0.0104 (13)	0.0009 (11)	0.0005 (10)	0.0050 (11)
O1	0.0210 (12)	0.0152 (13)	0.0131 (11)	−0.0027 (10)	−0.0055 (9)	0.0078 (10)
O2	0.0213 (12)	0.0181 (14)	0.0211 (13)	−0.0059 (10)	−0.0088 (10)	0.0084 (11)
C1	0.0165 (16)	0.0139 (17)	0.0142 (16)	−0.0020 (13)	−0.0026 (13)	0.0043 (14)
C2	0.0225 (17)	0.0125 (17)	0.0108 (15)	−0.0006 (13)	−0.0015 (13)	0.0058 (14)
C3	0.0226 (17)	0.0133 (17)	0.0139 (16)	−0.0012 (13)	0.0035 (13)	0.0076 (14)
C4	0.0133 (15)	0.0137 (17)	0.0145 (16)	0.0003 (13)	0.0029 (12)	0.0041 (14)
C5	0.0137 (15)	0.0115 (17)	0.0116 (15)	−0.0002 (12)	0.0019 (12)	0.0037 (13)
C6	0.0109 (15)	0.0113 (16)	0.0130 (15)	−0.0003 (12)	0.0006 (12)	0.0035 (13)
C7	0.0117 (15)	0.0106 (16)	0.0086 (14)	0.0021 (12)	−0.0001 (12)	0.0020 (13)
C8	0.0128 (15)	0.0144 (17)	0.0132 (15)	−0.0030 (13)	0.0006 (12)	0.0065 (14)
C9	0.0107 (15)	0.0173 (18)	0.0169 (16)	−0.0026 (13)	−0.0014 (13)	0.0052 (14)
C10	0.0117 (14)	0.0095 (16)	0.0090 (14)	0.0003 (12)	−0.0006 (12)	0.0024 (13)
C11	0.0123 (15)	0.0095 (16)	0.0127 (15)	0.0021 (12)	0.0040 (12)	0.0030 (13)
C12	0.0067 (14)	0.0132 (17)	0.0134 (15)	−0.0001 (12)	0.0000 (12)	0.0036 (13)
C13	0.0138 (15)	0.0134 (17)	0.0126 (15)	0.0019 (13)	−0.0006 (13)	0.0040 (14)
C14	0.0246 (17)	0.0163 (18)	0.0125 (16)	0.0001 (14)	−0.0046 (14)	0.0080 (14)

### Geometric parameters (Å, º)

**Table:**

I1—Cd	2.8765 (4)	C4—C5	1.397 (4)
I1—Cd^i^	2.9813 (3)	C4—H4	0.9500
I2—Cd	2.7023 (4)	C5—C6	1.471 (4)
Cd—N1	2.333 (2)	C6—H6	0.9500
Cd—N2	2.402 (2)	C7—C12	1.394 (4)
Cd—I1^i^	2.9813 (3)	C7—C8	1.399 (4)
N1—C1	1.332 (4)	C8—C9	1.388 (4)
N1—C5	1.346 (4)	C8—H8	0.9500
N2—C6	1.280 (4)	C9—C10	1.396 (4)
N2—C7	1.430 (4)	C9—H9	0.9500
O1—C13	1.342 (4)	C10—C11	1.395 (4)
O1—C14	1.452 (4)	C10—C13	1.491 (4)
O2—C13	1.207 (4)	C11—C12	1.390 (4)
C1—C2	1.396 (4)	C11—H11	0.9500
C1—H1	0.9500	C12—H12	0.9500
C2—C3	1.375 (5)	C14—H14A	0.9800
C2—H2	0.9500	C14—H14B	0.9800
C3—C4	1.394 (4)	C14—H14C	0.9800
C3—H3	0.9500		
			
Cd—I1—Cd^i^	92.716 (9)	C4—C5—C6	121.0 (3)
N1—Cd—N2	70.33 (8)	N2—C6—C5	120.1 (3)
N1—Cd—I2	107.66 (7)	N2—C6—H6	119.9
N2—Cd—I2	109.09 (6)	C5—C6—H6	119.9
N1—Cd—I1	134.10 (7)	C12—C7—C8	120.5 (3)
N2—Cd—I1	87.44 (6)	C12—C7—N2	117.5 (3)
I2—Cd—I1	117.588 (10)	C8—C7—N2	122.0 (3)
N1—Cd—I1^i^	86.88 (6)	C9—C8—C7	119.6 (3)
N2—Cd—I1^i^	141.81 (6)	C9—C8—H8	120.2
I2—Cd—I1^i^	106.827 (10)	C7—C8—H8	120.2
I1—Cd—I1^i^	87.284 (9)	C8—C9—C10	120.1 (3)
C1—N1—C5	118.7 (3)	C8—C9—H9	119.9
C1—N1—Cd	124.5 (2)	C10—C9—H9	119.9
C5—N1—Cd	116.87 (19)	C11—C10—C9	120.1 (3)
C6—N2—C7	119.8 (3)	C11—C10—C13	122.2 (3)
C6—N2—Cd	115.35 (19)	C9—C10—C13	117.6 (3)
C7—N2—Cd	124.74 (18)	C12—C11—C10	120.1 (3)
C13—O1—C14	114.2 (2)	C12—C11—H11	120.0
N1—C1—C2	122.7 (3)	C10—C11—H11	120.0
N1—C1—H1	118.6	C11—C12—C7	119.6 (3)
C2—C1—H1	118.6	C11—C12—H12	120.2
C3—C2—C1	118.8 (3)	C7—C12—H12	120.2
C3—C2—H2	120.6	O2—C13—O1	123.6 (3)
C1—C2—H2	120.6	O2—C13—C10	123.2 (3)
C2—C3—C4	119.0 (3)	O1—C13—C10	113.2 (3)
C2—C3—H3	120.5	O1—C14—H14A	109.5
C4—C3—H3	120.5	O1—C14—H14B	109.5
C3—C4—C5	118.8 (3)	H14A—C14—H14B	109.5
C3—C4—H4	120.6	O1—C14—H14C	109.5
C5—C4—H4	120.6	H14A—C14—H14C	109.5
N1—C5—C4	121.9 (3)	H14B—C14—H14C	109.5
N1—C5—C6	117.1 (3)		
			
Cd^i^—I1—Cd—N1	−83.00 (8)	Cd—N1—C5—C6	−5.0 (4)
Cd^i^—I1—Cd—N2	−142.16 (6)	C3—C4—C5—N1	3.0 (5)
Cd^i^—I1—Cd—I2	107.590 (12)	C3—C4—C5—C6	−175.7 (3)
Cd^i^—I1—Cd—I1^i^	0.0	C7—N2—C6—C5	179.1 (3)
N2—Cd—N1—C1	−175.4 (3)	Cd—N2—C6—C5	2.6 (4)
I2—Cd—N1—C1	−70.8 (3)	N1—C5—C6—N2	1.5 (5)
I1—Cd—N1—C1	119.0 (2)	C4—C5—C6—N2	−179.7 (3)
I1^i^—Cd—N1—C1	35.8 (3)	C6—N2—C7—C12	150.0 (3)
N2—Cd—N1—C5	4.5 (2)	Cd—N2—C7—C12	−33.8 (4)
I2—Cd—N1—C5	109.0 (2)	C6—N2—C7—C8	−32.9 (5)
I1—Cd—N1—C5	−61.1 (2)	Cd—N2—C7—C8	143.3 (2)
I1^i^—Cd—N1—C5	−144.3 (2)	C12—C7—C8—C9	0.8 (5)
N1—Cd—N2—C6	−3.7 (2)	N2—C7—C8—C9	−176.2 (3)
I2—Cd—N2—C6	−106.2 (2)	C7—C8—C9—C10	−0.7 (5)
I1—Cd—N2—C6	135.4 (2)	C8—C9—C10—C11	−0.8 (5)
I1^i^—Cd—N2—C6	53.1 (3)	C8—C9—C10—C13	175.7 (3)
N1—Cd—N2—C7	−180.0 (3)	C9—C10—C11—C12	2.2 (5)
I2—Cd—N2—C7	77.5 (2)	C13—C10—C11—C12	−174.0 (3)
I1—Cd—N2—C7	−40.9 (2)	C10—C11—C12—C7	−2.1 (5)
I1^i^—Cd—N2—C7	−123.2 (2)	C8—C7—C12—C11	0.6 (5)
C5—N1—C1—C2	1.5 (5)	N2—C7—C12—C11	177.7 (3)
Cd—N1—C1—C2	−178.6 (2)	C14—O1—C13—O2	4.4 (4)
N1—C1—C2—C3	1.7 (5)	C14—O1—C13—C10	−177.0 (2)
C1—C2—C3—C4	−2.5 (5)	C11—C10—C13—O2	156.5 (3)
C2—C3—C4—C5	0.2 (5)	C9—C10—C13—O2	−19.9 (5)
C1—N1—C5—C4	−3.9 (5)	C11—C10—C13—O1	−22.1 (4)
Cd—N1—C5—C4	176.3 (3)	C9—C10—C13—O1	161.5 (3)
C1—N1—C5—C6	174.9 (3)		

Symmetry code: (i) −*x*+1, −*y*+1, −*z*+1.

### Hydrogen-bond geometry (Å, º)

Cg1 is the centroid of the N1,C1–C5 ring.

**Table:**

*D*—H···*A*	*D*—H	H···*A*	*D*···*A*	*D*—H···*A*
C1—H1···O2^ii^	0.95	2.34	3.066 (4)	133
C14—H14*B*···*Cg*1^iii^	0.98	2.79	3.416 (4)	123

Symmetry codes: (ii) *x*+1, *y*, *z*+1; (iii) −*x*, −*y*+2, −*z*+1.
